# Editorial: Organ Fibrosis: Triggers, Pathways, and Cellular Plasticity

**DOI:** 10.3389/fmed.2016.00055

**Published:** 2016-11-15

**Authors:** Henricus A. M. Mutsaers, Peter Olinga

**Affiliations:** ^1^Department of Pharmaceutical Technology and Biopharmacy, University of Groningen, Groningen, Netherlands

**Keywords:** fibrosis, TGF-β, IFN-γ, chronic kidney disease, heme oxygenase-1, YAP/TAZ, macrophages

**The Editorial on the Research Topic**

**Organ Fibrosis: Triggers, Pathways, and Cellular Plasticity**

Pathological scar formation, i.e., fibrosis, is an integral part of the pathophysiological mechanism underlying organ failure and disease progression in a multitude of pathologies including Crohn’s disease, non-alcoholic steatohepatitis, and chronic kidney disease (CKD). As a consequence, fibrotic diseases account for up to 45% of deaths in the developed world (1). The fibrotic process is characterized by the excessive production and deposition of extracellular matrix (ECM) proteins, such as collagens, mainly by activated profibrotic fibroblasts. The disturbed equilibrium between ECM formation and degradation is the end result of a complex cascade of cellular and molecular responses initiated by organ damage, and even though there is a range of organ-specific triggers, the fibrotic process and associated signaling pathways are highly conserved between different organs. Although the cellular and molecular mechanisms driving fibrogenesis have been meticulously dissected in the last decades, novel insights into this complex pathological process keep arising. In this Research Topic, experts from all over the world delineate the latest findings pertaining to the diverse triggers, signaling pathways, and cell types associated with the induction and perpetuation of fibrogenic events.

As stated before, fibrosis can be induced by a variety of stimuli, some of which are non-specific and can affect all organs, e.g., inflammation, while other triggers are clearly associated with a specific pathology and organ, e.g., glomerular IgA deposition (2). The article by Simon and Hertig details how changes in fatty acid oxidation by transient acute kidney injury (AKI) can lastingly alter the epithelial features of renal proximal tubular cells. This change in phenotype promotes fibrogenesis, illustrating that AKI can be a cause of CKD and fibrosis (Simon and Hertig). In the review by Mutsaers et al., it is detailed how uremic retention solutes stimulate glomerular sclerosis as well as interstitial fibrosis. Furthermore, they describe that on a cellular level, uremic solutes induce fibrogenic events by promoting, among others, epithelial-to-mesenchymal transition and senescence (Mutsaers et al.). Regarding scar formation in the skin, Cremers et al. show that mechanical stress mitigated wound healing, which was associated with an enhanced inflammatory response and prolonged survival of fibroblast resulting in a profibrotic environment. Furthermore, they postulate that the cytoprotective enzyme heme oxygenase-1 is an interesting target for the treatment of fibrosis (Cremers et al.). These articles nicely reiterate the diversity of profibrotic triggers, ranging from the biochemical to the physical, indicating that there is still a plethora of potential therapeutic targets to be investigated in the coming years.

Historically, transforming growth factor (TGF)-β has been regarded as one of the key factors driving the fibrotic response in most organs. Binding of TGF-β to its receptor initiates a multifaceted response orchestrated by several SMAD proteins and signaling cofactors that dictate the final transcriptional response in a cell (3). However, as extensively described in the article by Piersma et al., the TGF-β pathway does not operate as a single entity but rather as part of an intricate signaling network composed of multiple signaling pathways including Wingless/Int (WNT) and yes-associated protein 1 (YAP)/transcriptional coactivator with PDZ-binding motif (TAZ) signaling, illustrating the complexity of fibrotic diseases.

Next to the diverse signaling pathways, a variety of (organ-specific) cells are involved in the fibrotic process including hepatic stellate cells (HSCs), renal pericytes, and circulating bone marrow-derived cells (4, 5). Interestingly, it appears that the latter cell type can acquire both a profibrotic as well as an antifibrotic phenotype as delineated in the article by Adhyatmika et al. Antifibrotic macrophages are tasked with the removal of fibrotic ECM and can be identified by the expression of, among others, matrix-degrading enzymes and collagen-uptake receptors. The concept of cell therapy for fibrosis with the notion that it is possible to identify and potentially recruit antifibrotic macrophages to fibrotic lesions foreshadows some exciting times to come.

Another interesting treatment modality revolves around the cytokine interferon gamma (IFNγ). The potent antifibrotic effects of IFNγ have been recognized for decades, yet this knowledge has never been successfully translated into a therapeutic application. The article by van Dijk et al. details the development of a construct that allows for HSC-specific delivery of IFNγ, thereby greatly improving the antifibrotic efficacy of this cytokine and reducing systemic side effects. This comprehensive review illustrates the feasibility of a targeted treatment for fibrosis, and we are curious to see how this research field will move forward.

All authors contributing to this Research Topic have made a tremendous effort to provide a scholarly overview of novel knowledge in the field of fibrosis research. It was a pleasure to work on this Topic, and we hope that the articles will provide the readers with a deeper understanding of fibrogenesis, thereby providing new vigor to the field and stimulating new avenues of research.

## Author Contributions

HM and PO conceived and wrote the manuscript. All the authors approved the final version of the manuscript and fully agreed with its content.

## Conflict of Interest Statement

The authors declare that the writing of this editorial was conducted in the absence of any commercial or financial relationships that could be construed as a potential conflict of interest.
